# Microbial diversity in the floral nectar of *Linaria vulgaris* along an urbanization gradient

**DOI:** 10.1186/s12898-016-0072-1

**Published:** 2016-03-30

**Authors:** Jacek Bartlewicz, Bart Lievens, Olivier Honnay, Hans Jacquemyn

**Affiliations:** Biology Department, Plant Conservation and Population Biology, KU Leuven, Kasteelpark Arenberg 31, 3001 Heverlee, Belgium; Laboratory for Process Microbial Ecology and Bioinspirational Management (PME and BIM), Department of Microbial and Molecular Systems (M2S), KU Leuven, Campus De Nayer, Fortsesteenweg 30A, 2860 Sint-Katelijne Waver, Belgium

**Keywords:** Nectar yeasts, Urbanization, *Metschnikowia*, *Acinetobacter*, Nestedness, Nectar microbial communities, *Linaria vulgaris*

## Abstract

**Background:**

Microbes are common inhabitants of floral nectar and are capable of influencing plant-pollinator interactions. All studies so far investigated microbial communities in floral nectar in plant populations that were located in natural environments, but nothing is known about these communities in nectar of plants inhabiting urban environments. However, at least some microbes are vectored into floral nectar by pollinators, and because urbanization can have a profound impact on pollinator communities and plant-pollinator interactions, it can be expected that it affects nectar microbes as well. To test this hypothesis, we related microbial diversity in floral nectar to the degree of urbanization in the late-flowering plant *Linaria vulgaris*. Floral nectar was collected from twenty populations along an urbanization gradient and culturable bacteria and yeasts were isolated and identified by partially sequencing the genes coding for small and large ribosome subunits, respectively.

**Results:**

A total of seven yeast and 13 bacterial operational taxonomic units (OTUs) were found at 3 and 1 % sequence dissimilarity cut-offs, respectively. In agreement with previous studies, *Metschnikowia reukaufii* and *M. gruessi* were the main yeast constituents of nectar yeast communities, whereas *Acinetobacter nectaris* and *Rosenbergiella epipactidis* were the most frequently found bacterial species. Microbial incidence was high and did not change along the investigated urbanization gradient. However, microbial communities showed a nested subset structure, indicating that species-poor communities were a subset of species-rich communities.

**Conclusions:**

The level of urbanization was putatively identified as an important driver of nestedness, suggesting that environmental changes related to urbanization may impact microbial communities in floral nectar of plants growing in urban environments.

## Background

Numerous studies have shown that microbes are common inhabitants of floral nectar [1–8]. Nectar inhabiting microbes (NIMs) have been shown to modify important physicochemical properties of floral nectar, such as sugar and amino acid composition [9, 10], to alter nectar odor [11, 12], and even to increase the temperature of the flower itself [13]. Changes in physicochemical properties of floral nectar can, in turn, alter the attractiveness of a given flower to pollinators, resulting in increased visitation rates and plant reproductive success [14, 15].

Most research so far has shown that microbial diversity in floral nectar is low and often dominated by a limited number of culturable species [4, 7, 8, 16, 17]. However, the few studies that have investigated variation in nectar microbial communities among plant populations have shown that the distribution of NIMs is not uniform, but can vary substantially between populations. For example, bacterial communities in the floral nectar of the summer asphodel (*Asphodelus aestivus*) changed significantly along an aridity gradient [18]. Similarly, microbial communities in the floral nectar of several populations of the spring-flowering forest herb *Pulmonaria officinalis* showed large within-population variation and low among-population similarity, indicating that NIM community assembly may to some extent be context-dependent [5]. These results further suggest that both variation in the local species pool of microbes and in local environmental conditions can shape NIM communities.

All studies investigating the diversity and abundance of NIMs so far have focused on plant populations occurring in natural environments, and virtually nothing is known about the composition of nectar microbial communities in urban environments. Transformation of natural landscapes by urbanization can, however, be expected to have a profound impact on nectar microbial communities. Given that most NIMs are vectored from one flower to the next by insect pollinators, and that nectar yeasts in particular are thought to rely exclusively on them to colonize new flowers [19, 20], impoverishment of pollinator communities can negatively affect colonization rates and subsequent dispersal of NIMs and therefore affect NIM community composition. The typically increasing cover of impervious surfaces in urban environments has, for example, been shown to be correlated with decreased nesting density in *Bombus vosnesenskii* and with decreased abundance of other wild bees [21, 22]. It has also been shown that different bee species respond differently to urbanization, which results in varied pollinator guilds along an urban gradient [23]. Furthermore, plant populations may typically decrease in size and become more spatially isolated as a result of urbanization. The increased spatial isolation and reduced population size of co-flowering plant species in urban environments can impede the exchange of microorganisms between populations, which, in turn, results in a nested species distribution pattern, where only the most common species are present in the most isolated or the smallest habitat patches (see [24] for review of the nestedness concept).

To test the general hypothesis that microbial communities in nectar change along an urbanization gradient, we investigated microbial communities in the floral nectar of the late-flowering herb *Linaria vulgaris* (yellow toadflax), a species that occurs in both urban and rural habitats. After collecting nectar from twenty populations across an urbanization gradient and surveying its microbiota using culture dependent methods and Sanger sequencing, we specifically addressed the following questions:

1. How does urbanization affect microbial incidence in floral nectar of *L. vulgaris*?
2. Does urbanization lead to impoverished NIM communities, with the most heavily urbanized sites having the most impoverished communities?
3. Are impoverished NIM communities subsets of more species-rich communities?

## Results and discussion

### Yeast diversity and occurence

**Fig. 1:**
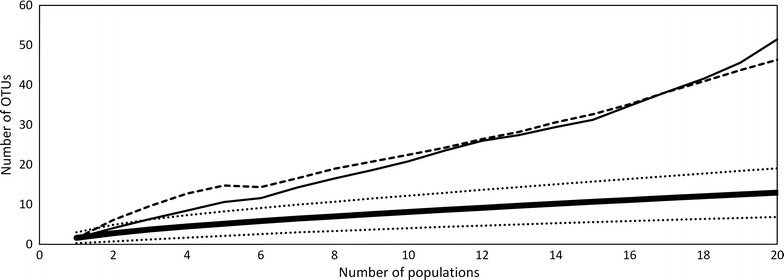
Rarefaction curve (*bold*, *solid line*) and the estimators Chao2 (*thin solid line*) and ICE (*dashed line*) of yeast operational taxonomic unit (OTU) richness found in the floral nectar of 20 *Linaria vulgaris* populations sampled across an urbanization gradient in the vicinity of Leuven, Belgium. The sequences were grouped into OTUs based on 97 % identity at the large ribosomal subunit gene

Following cultivation and isolation a total of 140 yeast isolates was obtained. When the sequences corresponding to these isolates were clustered according to a 3 % dissimilarity cutoff, seven fungal OTUs were identified (Table 1). Following BLAST analysis, these OTUs corresponded to five different validly named species from four families: *Metschnikowiaceae*, *Dothioraceae*, *Sporidiobolales* and *Tremellales*, indicating that *Ascomycota* were more frequently represented than *Basidiomycota*. Most isolates were related to *Metschnikowia gruessi* (101 isolates) and to a smaller degree to *M. reukaufii* (36 isolates), while other species were represented by single isolate (Table 1). Three out of the seven OTUs (OTU_0.03_Y2, OTU_0.03_Y3, and OTU_0.03_Y4) found were identified as *M. reukaufii*. The Chao2 estimator predicted a slightly higher number of OTUs, 12.7, while the ICE estimator predicted 18.61 OTUs (Fig. 1).

**Table 1:** Yeast operational taxonomic units (OTUs) identified in the nectar of 20 *L. vulgaris* populations sampled along an urbanization gradient

OTU 3 %	118	Phylum	Family	Closest match with GenBank entries	Accession number	Score	E value	Length	Identity (%)
OTU_0.03_Y1	22	Ascomycota	Metschnikowiaceae	*Metschnikowia gruessi*	JX067745	665.914	0	360	100
OTU_0.03_Y2	10	Ascomycota	Metschnikowiaceae	*Metschnikowia reukaufii*	KM281795	682.534	0	369	100
OTU_0.03_Y3	4	Ascomycota	Metschnikowiaceae	*Metschnikowia reukaufii*	FJ455114	675.147	0	365	100
OTU_0.03_Y4	1	Ascomycota	Metschnikowiaceae	*Metschnikowia reukaufii*	JN642530	584.662	3.18e^−163^	332	98.5
OTU_0.03_Y5	1	Ascomycota	Dothioraceae	*Aureobasidium pullulans*	KP710217	774.886	0	419	100
OTU_0.03_Y6	1	Basidiomycota	Sporidiobolales	*Sporobolomyces roseus*	AM160644	250.48	1.32e^−62^	135	100
OTU_0.03_Y7	1	Basidiomycota	Tremellales	*Cryptococcus aureus*	JN004200	1059.25	0	583	99.5

The sequences were grouped into OTUs based on 97 % identity at the large ribosomal subunit gene. The BLAST search was conducted in June 2015, excluding uncultured/environmental samples. Only the closest matches are reported

Yeasts were found in all studied *L. vulgaris* populations. On average, there were two yeast OTUs present per population (sd: 0.79), with *M. gruessi* present in 95 % of the populations, and *M. reukaufii* present in 70 % of the populations. There was no correlation between yeast incidence and ISI_500_ (Spearman-*ρ* = 0.06, *p* = 0.78) or plant population size (Spearman-*ρ* = 0.02, *p* = 0.98). The size of each population and impervious surface index pertaining to it were not inter-correlated (Spearman-*ρ* = −0.32, *p* = 0.15).

Thus, our results showed that yeast communities in urban environments were mainly dominated by the yeasts *M. reukaufii* and *M. gruessi*. However, unlike in many natural environments [17, 25–27] *M*. *gruessi* was more abundant than *M. reukaufii*. Assessment of the phenotypic landscape of both yeast species has recently shown that both species displayed a significantly different physiological profile, most likely facilitating co-occurrence of both species [28]. Moreover, comparison of utilization profiles in single vs mixed cultures indicated that *M. reukaufii* generally grows better in sucrose solutions, and that *M. gruessi* grows better in mixed cultures in glucose and fructose solutions [28]. On the other hand, out of the yeast strains isolated from 19 different nectars, the strains isolated from the nectar of *Plantaginaceae*, the family to which *L. vulgaris* belongs, exhibited the most dissimilar phenotypes for *M. gruessi* and *M. reukaufii*. This might mean that the nectar of Plantaginaceae exhibits strong differential constraints on yeast species growth, with which *M. gruessi* copes better in the specific case of *L. vulgaris.*

### Bacterial diversity and occurence

**Fig. 2:**
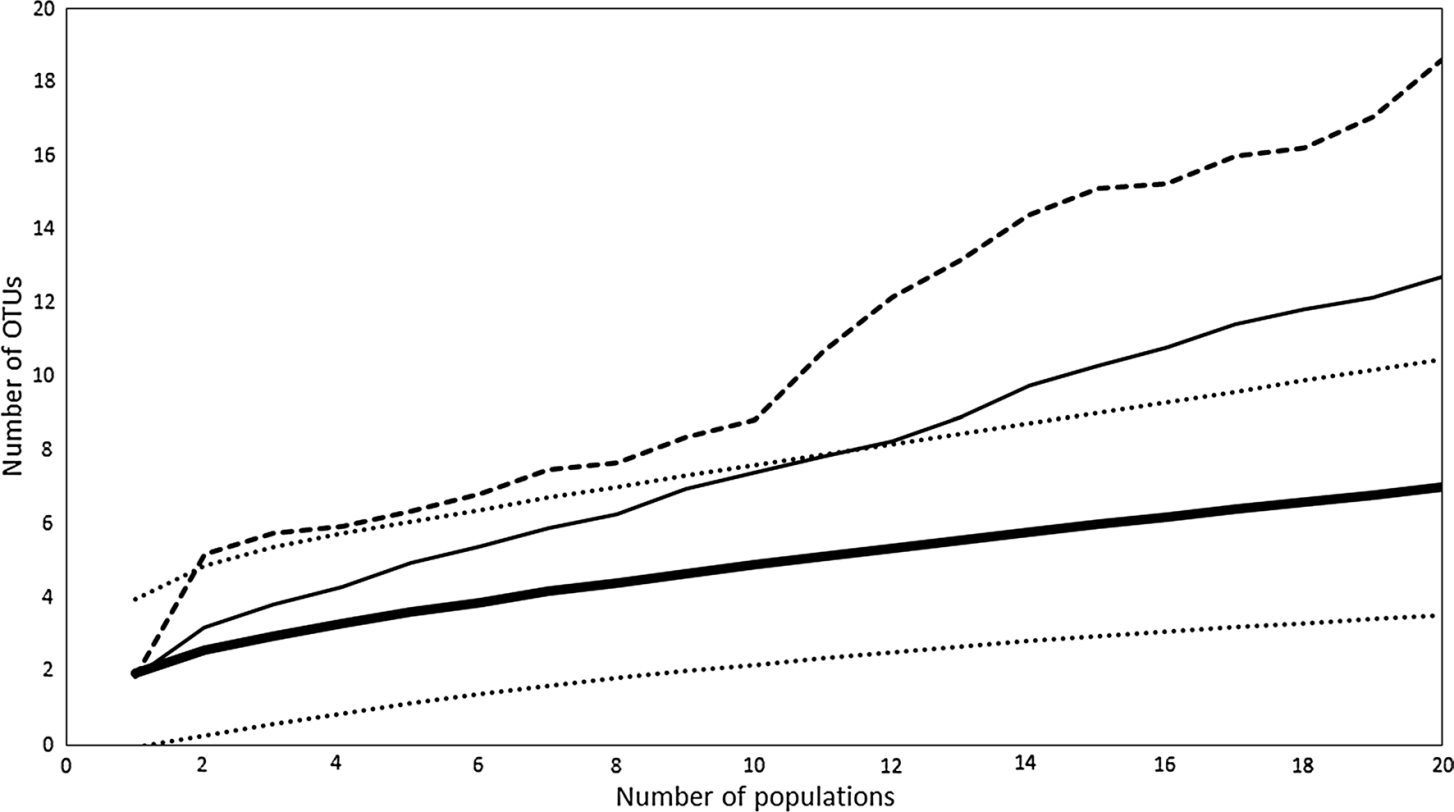
Rarefaction curve (*bold*, *solid line*) and the estimators Chao2 (*thin solid line*) and ICE (*dashed line*) of bacterial operational taxonomic unit (OTU) richness found in the floral nectar of 20 *Linaria vulgaris* populations sampled across an urbanization gradient in the vicinity of Leuven, Belgium. The sequences were grouped into OTUs based on 99 % identity at the small ribosomal subunit gene

In total, 52 bacterial isolates were obtained after cultivation. Thirteen bacterial OTUs were identified when clustered according to 1 % dissimilarity cutoff (Table 2). Based on BLAST results, these OTUs corresponded to 13 validly named species. The ICE and Chao2 estimators predicted a much higher number of 46.37 and 51.48 OTUs, respectively (Fig. 2). Similarly, the rarefaction curves showed that additional sampling would yield a higher number of OTUs, and this effect was more pronounced than in the case of yeast, probably due to higher number of bacterial singletons. The rarefaction curves for bacteria showed a similar level of saturation, however, to what has been found in earlier culture-dependent studies (i.e. 4, 5, 20). *Acinetobacter nectaris* comprised over 50 % (29 isolates) of bacterial isolates, with *Rosenbergiellaepipactidis* coming second (10 isolates). The remaining OTUs were represented by 1–3 isolates, coming mostly from the *Enterobacteriaceae*, but also from *Pseudomonadaceae*, *Microbacteriaceae*, *Bacillaceae* and *Sphingomonadaceae* (Table 2).

**Table 2:** Bacterial operational taxonomic units (OTUs) identified in the nectar of 20 *L. vulgaris* populations sampled along an urbanization gradient

OTU 1 %	Number of isolates	Phylum	Family	Closest match with GenBank entries	Accession number	Score	E value	Length	Identity (%)
OTU_0.01_B1	1	Actinobacteria	Microbacteriaceae	*Microbacterium testaceum*	KP642087	1796.06	0	972	100
OTU_0.01_B2	1	Actinobacteria	Microbacteriaceae	*Rathayibacter festucae*	NR_042574	1768.36	0	957	100
OTU_0.01_B3	1	Firmicutes	Bacillaceae	*Lysinibacillus odysseyi*	NR_113881	1921.64	0	1043	100
OTU_0.01_B4	1	Proteobacteria	Sphingomonadaceae	*Sphingomonas faeni*	KM891564	1773.9	0	960	100
OTU_0.01_B5	1	Proteobacteria	Enterobacteriaceae	*Pantoea ananatis*	KC139412	1919.79	0	1048	99.7
OTU_0.01_B6	1	Proteobacteria	Enterobacteriaceae	*Pantoea vagans*	KP099965	1757.28	0	957	99.8
OTU_0.01_B7	1	Proteobacteria	Enterobacteriaceae	*Pectobacterium carotovorum*	GU129979	1628.02	0	885	99.9
OTU_0.01_B8	1	Proteobacteria	Enterobacteriaceae	*Ewingella americana*	KM891553	1842.23	0	997	100
OTU_0.01_B9	1	Proteobacteria	Pseudomonadaceae	*Pseudomonas viridiflava*	NR_117825	1899.48	0	1028	100
OTU_0.01_B10	3	Proteobacteria	Pseudomonadaceae	*Pseudomonas moraviensis*	KP165022	1892.09	0	1024	100
OTU_0.01_B11	10	Proteobacteria	Enterobacteriaceae	*Rosenbergiella epipactidis*	NR_126303	1816.38	0	989	99.8
OTU_0.01_B12	1	Proteobacteria	Moraxellaceae	*Acinetobacter boissieri*	NR_118409	1855.16	0	1004	100
OTU_0.01_B13	29	Proteobacteria	Moraxellaceae	*Acinetobacter nectaris*	JQ771134	1879.16	0	1023	99.8

The sequences were grouped into OTUs based on 99 % identity at the small ribosomal subunit gene. The BLAST search was conducted in June 2015, excluding uncultured/environmental samples. Only the closest matches are reported

Bacteria were present in all but four of the studied populations, with an average of 1.65 OTUs per population (sd: 1.26). *A. nectaris* was the most frequently found species and occurred in 60 % of the populations, while *R.epipactidis* was present in 35 % of the populations. Bacterial incidence was also not correlated to ISI_500_ (Spearman-*ρ* = −0.16, *p* = 0.49), but it was significantly correlated to plant population size (Spearman-*ρ* = 0.45, *p* = 0.04) (Table 3).

**Table 3:** Properties of the 20 sampled* Linaria vulgaris* populations

Population	Longitude	Latitude	ISI_500_ (%)	Population size	Nestedness rank	Yeast OTUs_0.03_ richness	Total yeast incidence (%)	Bacterial OTUs_0.01_ richness	Total bacterial incidence (%)	Total microbial richness
A	4.709365	50.864275	36.7	400	3	3	100	3	100	6
B	4.704492	50.863418	33.84	450	20	2	100	0	0	2
C	4.700154	50.86323	42.35	100	14	2	100	1	40	3
D	4.676421	50.885377	44.85	15	18	2	20	0	0	2
E	4.715524	50.867437	27.44	60	13	2	60	0	0	2
F	4.713976	50.85837	18.9	520	11	2	80	2	60	4
G	4.72284	50.850707	44.72	420	19	2	100	0	0	2
H	4.7281	50.843644	39.17	300	15	2	40	1	80	3
I	4.723781	50.852871	32.26	70	12	1	60	2	80	3
J	4.685015	50.888567	31.16	150	4	1	40	4	80	5
K	4.725668	50.858904	19.26	200	17	1	100	1	40	2
L	4.727276	50.861677	22.98	83	6	3	80	2	40	5
M	4.725735	50.860678	19.78	500	2	3	80	2	100	5
N	4.801452	50.822292	7.06	500	7	2	60	3	40	5
O	4.803312	50.823027	12.99	500	16	1	40	1	20	2
P	4.784239	50.826077	7.81	170	9	2	60	1	40	3
Q	4.79446	50.825404	5.98	400	10	1	40	2	60	3
R	4.790337	50.825352	6.65	1250	1	3	80	3	80	6
S	4.794894	50.823779	6.99	160	5	1	100	4	80	5
T	4.805983	50.852606	5.36	50	8	3	80	1	20	4

The approximate coordinates of each population are given in decimal degrees. The degree or urbanization of the surroundings of each population was assessed using ISI_500_, the impervious surface index within a radius of 500 m. For each of the sampled populations, the bacterial and yeast species richness (number of OTUs) and incidence (frequency of occurrence) are presented

Overall, the incidence of bacteria in *L. vulgaris* (which were present in 48 % of the nectar samples) was higher than the previously reported average of 19.9 % for Mediterranean species, the 6.5 % for *Linaria viscosa* in Spain [29], and closer to the 53.5 % reported from South African plant species [4]. Interestingly, we found a high incidence of the recently described bacterial species *A. nectaris* [29]. This bacterium was found originally in the nectar of Mediterranean plant communities [4]. However, our results indicate that it is not solely restricted to the Mediterranean area, but that it can also be found frequently in the floral nectar of plants growing in North-Western Europe. *A. nectaris* was previously isolated in Belgium in some orchid species, but by no means was it a nectar dominating bacterial species [6]. In another study on nectar microbial communities of *P. officinalis* in Belgium, it was not found either [5]. In Israel, Fridman et al. [30] found bacteria belonging to the *Acinetobacter* genus in at least half of the samples from each of the three Mediterranean plant species they studied. *A. nectaris* was later detected in nectar of *Asphodelus aestivus* in the same country [18]. This suggests that despite its broad distributional range, it might be plant species specific, either due to the nectar properties it requires, or due to its introduction route, which is unknown so far. It is noteworthy, however, that of the two recently described nectar specialist *Acinetobacter* taxa found in this study, *A. nectaris* was represented by 29 isolates, but *A. boissieri*, only by one isolate, which parallels the situation in the *Metschnikowia* genus, when one or the other of the two nectar specialist yeasts is usually dominant in any given plant species.

The second most abundant bacterium inhabiting the nectar of *L. vulgaris* was *R. epipactidis,* also a species previously isolated from nectar [31, 32]. Perhaps its lower abundance could be explained by its less effective dissemination method: it is speculated that thrips serve as its vectors [18]. Interestingly, although bacterial-yeast co-occurrence was common at the population level, it was less so at the plant level. This could be due to priority effects and that nectar colonized by yeasts could be less accessible to bacteria. However to confirm this, further co-occurrence studies on the nectary level are needed, as well as competition experiments [33].

### Spatial distribution of yeasts and bacteria

**Fig. 3:**
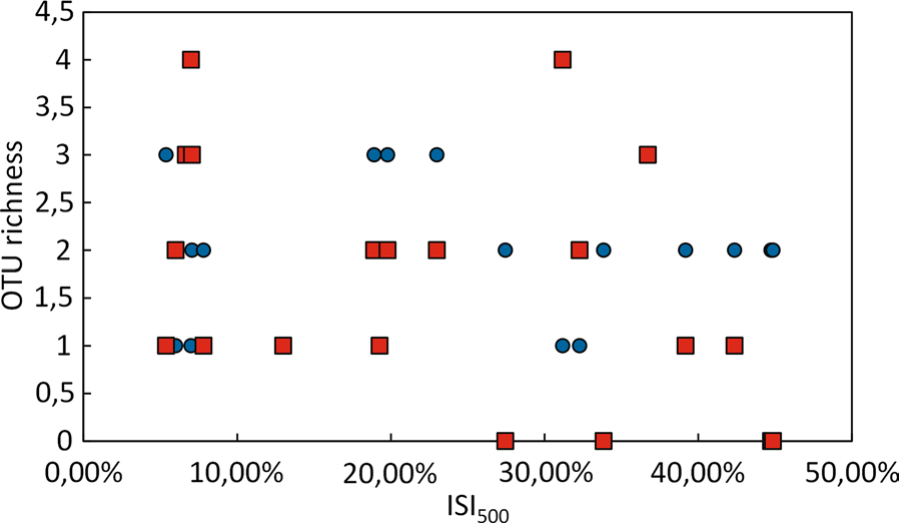
A scatter plot of urbanization versus microbial OTU richness found in the nectar of the 20 *Linaria vulgaris* populations located across an urbanization gradient in the vicinity of Leuven, Belgium. Urbanization was measured as an impervious surface percentage within a 500 m radius from each sampled population. Bacterial OTUs were defined as sequence clusters exhibiting at least 1 % dissimilarity from each other, and presented red squares while yeast OTUs were defined as sequence clusters exhibiting at least 3 % dissimilarity from each other, and presented as blue circles. There was no correlation between bacterial nor yeast OTU richness and urbanization

Yeast OTU richness, measured as the number of OTUs per plant population, was not related to urbanization level (Spearman-*ρ* = 0, *p* = 1.00) nor to population size (Spearman-*ρ* = 0.005, *p* = 0.98) (Fig. 3). The number of bacterial OTUs in each *L. vulgaris* population was also not related to plant population size (Spearman-*ρ* = 0.30, *p* = 0.19), but was marginally negatively correlated to the ISI_500_ pertaining to this population (Spearman *ρ* = −0.43, *p* = 0.056) (Fig. 3). When bacterial and yeast OTU numbers were combined, the correlation with ISI_500_ was not significant (Spearman-*ρ* = −0.36, *p* = 0.11).

**Fig. 4:**
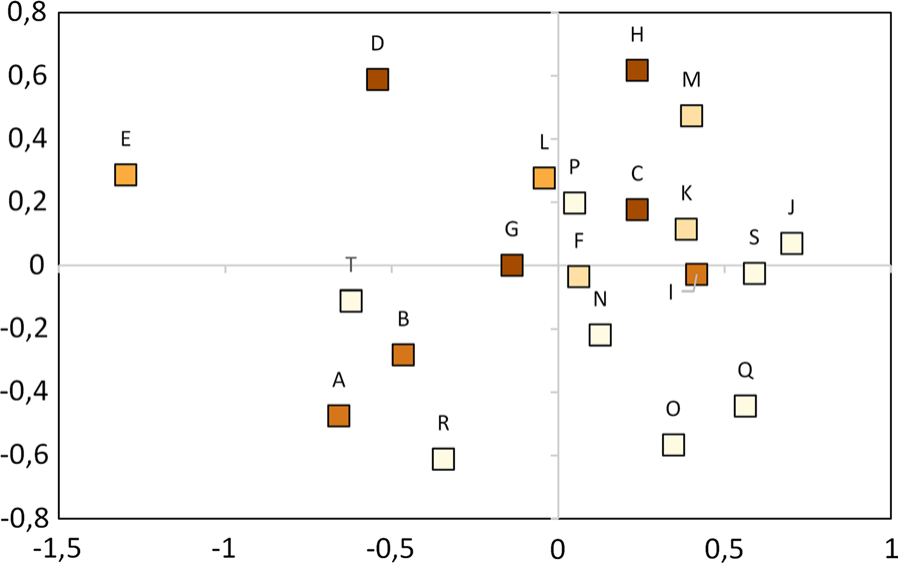
NMDS ordination of the total microbial community composition (bacteria and yeasts) in the floral nectar of 20 *Linaria vulgaris* populations located across an urbanization gradient in the vicinity of Leuven, Belgium. The populations have been color coded to represent the degree of impervious surfaces within 500 m radius of each population, as in Fig. 7

**Fig. 5:**
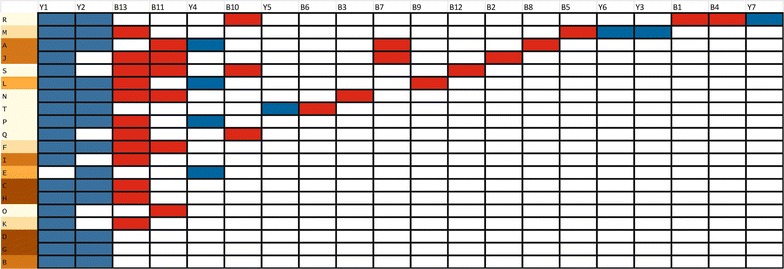
Matrix of incidence of nectar microbial communities in the 20 *Linaria vulgaris* populations located across an urbanization gradient in the vicinity of Leuven, Belgium.* Rows* are populations and* columns* are OTUs. The populations have been color coded to represent the degree of impervious surfaces within 500 m radius of each population, as in Fig. 7. The matrix was sorted according to ‘binmatnest’ algorithm as implemented in the R package ‘bipartite’. The presence of yeast communities is indicated in *blue*, while presence of bacterial communities is indicated in *red*. The communities showed significant nestedness (*T* = 16.81, *p* < 0.01, NODF = 42.19, *p* < 0.01), and their nestedness rank was correlated with impervious surface index (Spearman-ρ = 0.49, *p* = 0.02), which implies that nectar urban microbial communities are a subset of nectar rural microbial communities

**Fig. 6:**
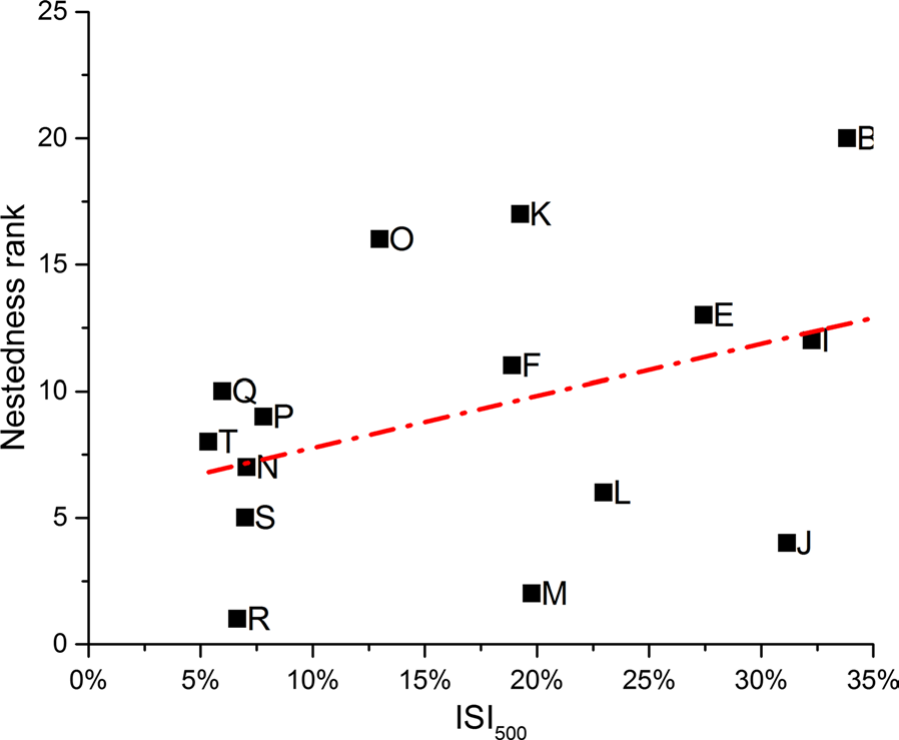
A scatter plot of urbanization versus nestedness rank of communities inhabiting each of the 20 *Linaria vulgaris* populations located across an urbanization gradient in the vicinity of Leuven, Belgium. Urbanization was measured as an impervious surface percentage within a 500 m radius from each sampled population. The nestedness rank was assigned according to ‘binmatnest’ algorithm as implemented in the R package ‘bipartite’. Urbanization and nestedness rank of microbial communities showed significant correlation (Spearman-ρ = 0.49, *p* = 0.02)

Co-occurrence of bacteria and yeasts was common at the population level, with all populations harboring yeasts, and only four lacking bacteria. A smaller degree of co-occurrence was observed at the plant level, where yeast and bacteria were found together in 33 % of the samples. A Mantel test showed no correlation between geographic distance and community dissimilarity for neither bacterial and yeast community composition separately (r = −0.08, *p* = 0.83 and r = 0.03, *p* = 0.34, respectively), nor when they were combined (r = −0.08, *p* = 0.82), NMDS also revealed no apparent clustering (Fig. 4). NMDS axis 1 was not correlated to ISI_500_ (Spearman-*ρ* = 0.2, *p* = 0.39), but NMDS axis 2 was (Spearman-*ρ* = 0.47, *p* = 0.03). Finally, the NIM communities showed significant nestedness (*N* = 0.83, *p* < 0.01; NODF = 42.19, *p* < 0.01), implying that species-poor communities were a subset of species-rich communities (Fig. 5). The nestedness rank was significantly correlated to ISI_500_ (Spearman-*ρ* = 0.49, *p* = 0.02), but not to plant population size (Spearman-*ρ* = −0.18, *p* = 0.42) (Fig. 6) . The nestedness rank correlation with ISI_500_ has to be treated with caution, since the rarefaction curves were not completely saturated, especially for bacteria (Figs. 1, 2). Additional OTUs would likely appear if the number of samples increased.

In contrast to our hypothesis, we did not find any clear changes in microbial incidence per se in the nectar of *L. vulgaris* across the studied urbanization gradient. OTU richness of urban and non-urban nectar communities was similar for yeasts, whereas urban bacterial nectar communities were marginally poorer than non-urban ones. Similarity in community composition did also not decrease with geographic distance, and no apparent clustering was detected, suggesting that the species pools from which NIMs colonize their nectary microhabitats are homogeneously throughout the area. These results further suggest that pollinators were still present in urban environments in sufficient numbers to effectively disperse bacteria and yeast. Moreover, the most urbanized plant populations we studied were still located in sites with only 45 % of impervious surface coverage within 500 m. It is therefore not impossible that the effect of such moderate urbanization on pollinator guilds is not drastic, and that microbial frequency would decrease sharply in more extremely urbanized areas.

**Fig. 7:**
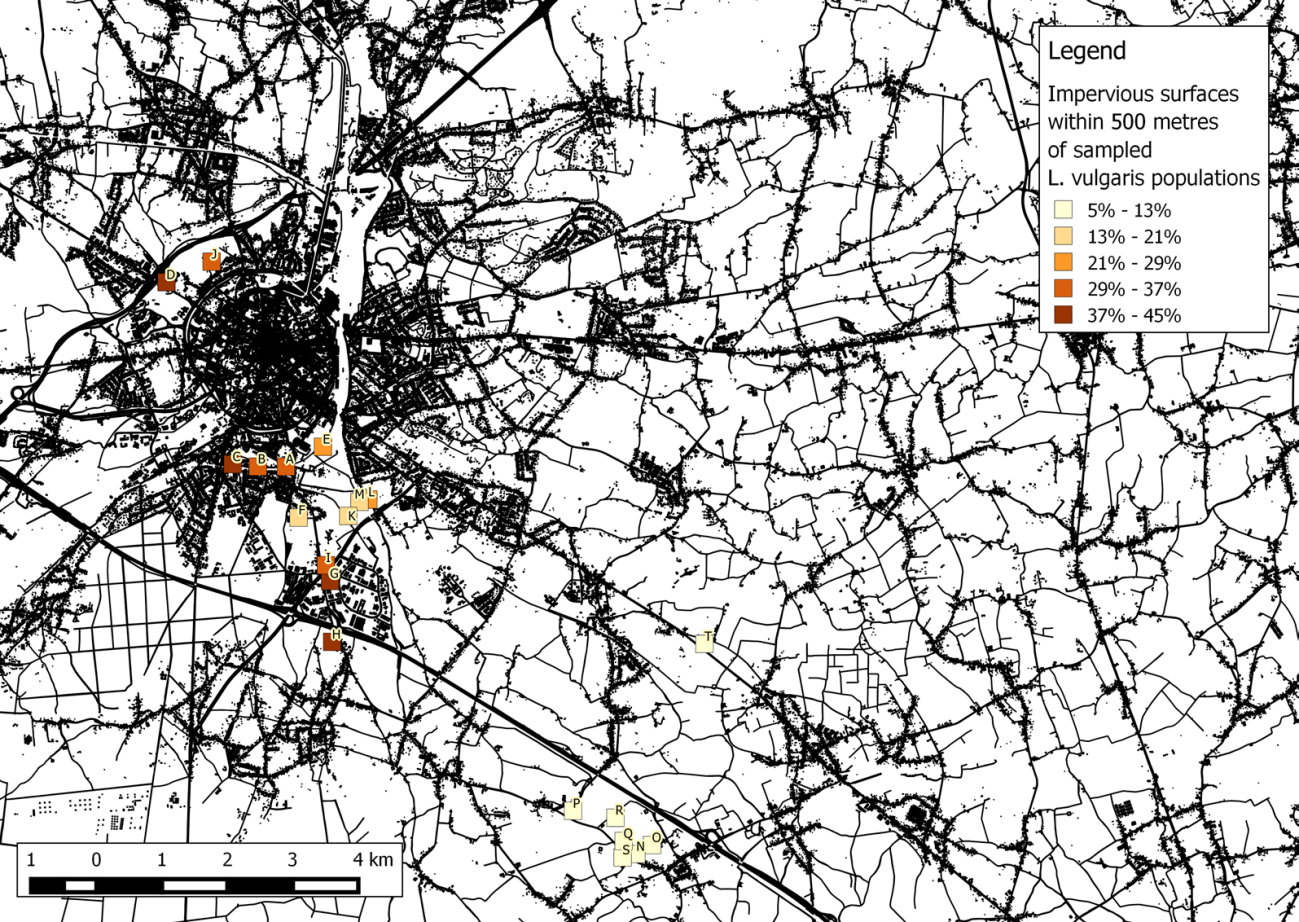
Map showing the locations of the 20 *Linaria vulgaris* populations studied in the vicinity of Leuven, Belgium. These populations have been color coded to represent the degree of impervious surfaces within 500 m radius of each population. From these populations, five shoots were chosen at random, and a single inflorescence was taken from each plant. Nectar was collected from four flowers belonging to each inflorescence

On the other hand, the studied communities showed significant nestedness, indicating that OTU-poor communities were a subset of OTU-rich communities, and that generalist species occurred everywhere and specialist species were restricted to particular populations. The significant albeit weak correlation between urbanization and nestedness rank, and the lack of such correlation with plant population size may imply that the urban communities were a subset of the rural communities. Because many of the *L. vulgaris* populations located in sites with very low impervious surface coverage within 500 m were geographically relatively close to each other (Fig. 7), and because rarefaction analyses revealed potentially incomplete sampling, this correlation could be a result of some unknown spatial effect unrelated to urbanization. However, such correlation could also suggest that populations in urban environments may be less frequently visited by pollinators due the higher isolation of *Linaria* populations in urban environments or the lack of other co-flowering plant species that attract pollinators, which in turn limits the exchange of less prevalent species. Alternatively, these results may suggest that nectar conditions in urban environments are less suitable for microbial growth than in natural environments and that only generalist species are capable of growing in the floral nectar of plants growing in urban environments. This result is consistent with the hypothesis that nectar yeasts should be capable of exploiting a wide range of nectar microhabitats, as they rank as two first entries in the nestedness matrix (Pozo et al. [17]). Interestingly, most bacteria, including nectar specialists, rank further in the nestedness matrix, which suggests that they are less adapted to different nectar types than yeasts.

## Conclusion

Overall, our results show that nectar yeasts are able to grow well in anthropogenically transformed environments. They grow equally well in autumn-flowering species *L. vulgaris* as in species that bloom earlier in the season on which previous studies were focused: *Helleborus foetidus*, *Pulmonaria officinalis*, *Delphinium nuttalianum* or *Asphodelus aestivus*. Moreover, our results indicate that not only nectar yeast communities, but also nectar bacterial communities are species poor and dominated by two specialist genera that seem to have a broad geographic range. Although urbanization did not seem to affect overall microbial richness nor incidence, microbial communities inhabiting the nectar of urban populations of *L. vulgaris* were nested in the communities inhabiting the nectar of its rural populations, although this result has to be treated with caution due to sampling limitations. Nevertheless, this might mean that urbanization may increase the isolation of plant populations harboring nectar microhabitats, resulting in reduced prevalence of rare species. Further research in this field will need to take into account two aspects that were outside of the scope of this study. Firstly, visitation rates, community composition and abundance of pollinators will have to be established more precisely in the chosen study system to give reliable estimations of their influence on nectar communities. Secondly, it is known that but a fraction of microbes can be successfully cultivated [34]. This is also reflected by the fact that rarefaction curves in studies relying on culture dependent methods are frequently not saturated, which decreases the level of confidence one can put into conclusions drawn from OTU richness and diversity. Perhaps more pronounced effects of urbanization could be detected with a more sensitive technique to measure their community composition, for instance through next-generation sequencing.

## Methods

### Study species and study area

*Linaria vulgaris* is a self-incompatible, perennial herb that is characterized by an extensive root system. It produces zygomorphic, bright yellow flowers with an orange nectar guide and a long spur between summer and late autumn [35]. The species is mainly pollinated by long-tongued bumblebees, such as *B. hortorum* and *B. pascuorum* [36, 37]. Short-tongued species, such as *B. terrestris*, behave like nectar robbers and access nectar illegitimately by biting a hole in the spur of a *Linaria* flower [37]. The main sugar found in *L. vulgaris* nectar is sucrose, but glucose, fructose and even trace amounts of raffinose have also been found [35].

Twenty *L. vulgaris* populations that grew in locations exhibiting different levels of urbanization were examined (Table 3). No sampling permissions were required to access the populations and sample them. The study area consisted of the city of Leuven (situated about 25 km east of Brussels, Belgium) and its immediate suburbs that gradually shift into areas with a more rural character. To assess the level of urbanization of the locations where each *L. vulgaris* population grew, for every of these locations we calculated an impervious surface index within a radius of 500 m (ISI_500_), as described previously [38] to serve as a proxy for urbanization (Fig. 7). In some cases, the 500 m radii overlapped. Some suburban areas also showed considerable amount of impervious surfaces within the 500 m radius, and no suitable populations were found in a 3 km stretch between populations P and H. Populations T, D and J were located outside of a linear transect encompassing all of the other populations. Given these deviations, our design was an approximation of sampling along an idealized urban–rural gradient. For each population, we also assessed its size by counting the number of inflorescences. Although the number of inflorescences does not necessarily reflect the total number of genets in the population, it does reflect the number of flowers that is perceived by pollinators.

### Sampling and cultivation of microorganisms

From each population, five plants were randomly chosen, and a single inflorescence per plant was brought to the laboratory for further processing on the same day. Given that NIMs are often patchily distributed among flowers within an inflorescence [39], nectar from four flowers from one inflorescence was pooled. Within 8 h of collection, nectar was harvested. To this end, the spur was first cut with a sterile scalpel in order to avoid pollen contamination, and then 0.5 µl nectar was extracted using a micropipette. A total of 2 µl, pooled from four flowers, was diluted in 98 µl of water and kept at 4 °C. Within 24 h, 2 µl of the diluted nectar was plated on tryptone soy agar (TSA) supplemented with 0.01 % chloramphenicol or 0.01 % actidione in order to suppress the growth of bacteria and yeasts, respectively. The plates were incubated at 25 °C for 7 days. Subsequently, one colony of each morphotype per plate was isolated and purified on TSA. It has been shown in previous studies on nectar microbiota that identical morphotypes generally correspond to identical species [5, 6]. After purification, each isolate was again transferred to TSA after which it was stored at −80 °C in 96 well plates containing 40 % glycerol.

### DNA extraction

Genomic DNA was extracted according to the procedure described by Pozo et al. [17]. Subsequently, partial ribosomal RNA genes were amplified using the primer pairs 27F/1492R and NL1/NL4 for bacteria and yeasts, respectively [4, 40]. When amplification failed using 27F/1492R, the forward primer 63F [41] was used instead of 27F. PCR amplification was performed using a Bio-Rad T100 thermal cycler in a reaction volume of 20 µl containing 0.5 µM of each primer, 0.25 mM of each dNTP, 1.25 units Takara Taq DNA polymerase, 1 × Takara Taq PCR buffer (Clontech Laboratories, Palo Alto, CA, USA), and 5 ng genomic DNA (as measured by a Nanodrop spectrophotometer). The reaction mixture was initially denatured at 94 °C for 2 min, followed by 35 cycles of 45 s at 94 °C, 45 s at 55 °C (yeasts) or 59 °C (bacteria), and 45 s at 72 °C, with a final extension at 72 °C for 10 min. Subsequently, obtained amplicons were sequenced by Macrogen Inc. using the same reverse primers as those used for amplification.

### Data analysis

Obtained sequences were individually trimmed for quality, using a minimum Phred score of 20, and, in cases of ambiguous base calls, manually edited based on the obtained electropherograms. Subsequently, each sequence was assigned a taxonomic identity based on BLAST [42] results using the GenBank nucleotide (nt) database [43], excluding environmental/uncultured samples. Next, the bacterial and yeast sequences were separately aligned using the MUSCLE algorithm implemented in Geneious 7R. These alignments have been used to create genetic distance matrices, which served as a basis to assign the sequences to OTUs using Mothur v 1.32.1 at 1 % and 3 % dissimilarity cutoffs for bacteria and yeasts, respectively. For each OTU, a representative sequence as determined by Mothur has been deposited in GenBank (accession numbers: KT347518–KT347530 for bacteria and KU900119–KU900125 for yeast).

Using EstimateS v 9.1.0, rarefaction analyses were conducted to assess our sampling effort. EstimateS was also used to calculate the ICE and Chao2 estimators of species richness [44]. For each *L. vulgaris* population, OTU richness and incidence were calculated. Incidence in a given population was expressed as percentage of samples that contained microorganisms. OTU richness was defined as the total number of OTUs per given population. Both of these variables were related to the urbanization measure ISI_500_ and plant population size using a Spearman rank correlation.

To visualize differences in microbial community composition among populations, we applied the non-metric multidimensional scaling (NMDS) ordination technique using the vegan package [45] in R software. As distance measure, we used the Bray-Curtis coefficient. EstimateS software was used to create a dissimilarity in community composition matrix, using the Jaccard estimator. A Mantel test using the R software was then conducted to relate the dissimilarities in community composition with geographical distances between populations. The significance of the Mantel test was assessed by performing 9999 permutations.

Lastly, we tested the hypothesis that NIM communities were significantly nested, i.e. that species-poor communities were a subset of the more rich ones and that rare OTUs were only present in the most OTU rich communities [46–48]. Two different measures to estimate the degree of nestedness were applied. We first calculated *N* = (100−*T*)/100, where *T* is the matrix temperature, a measure of matrix disorder that varies between 0° (perfectly nested) and 100° (perfectly non-nested). Values of *N* close to one thus indicate a high degree of nestedness. However, because *T* may be dependent on the size and shape of the species presence matrix, we also calculated a second nestedness measure, based on overlap and decreasing fill (NODF), correcting for these flaws [48]. Conversely to *T*, high values of NODF signify a nested matrix structure.

Two different null models implemented in ANINHADO were used to test the significance of nestedness [47]. In the first null model, each cell in the interaction matrix has the same probability of being occupied. This null model is very general and does not take into account the fact that the number of connections per species may vary substantially. A more conservative null model would therefore be a model in which the probability of drawing an interaction is proportional to the degree of specialization [49]. In this null model, the probability of each cell being occupied is the average of the probabilities of occupancy of its row and column [48]. Finally, to identify the potential drivers behind the nestedness of our dataset, a nestedness rank was assigned to each sampling site according to the ‘binmatnest’ algorithm in the R package *bipartite*. Afterwards, plant population size and ISI_500_ was related to nestedness rank using the Spearman rank correlation.

## Abbreviations

BLAST: basic local alignment search tool
ISI_500_: impervious surface index within 500 m radius
OTU: operational taxonomic unit
NIMs: nectar inhabiting microbes
NMDS: non metric multidimensional scaling
NODF: nestedness metric based on overlap and decreasing fill
PCR: polymerase chain reaction
TSA: tryptone soya agar
